# Cultural Dimensions of Depression in Bangladesh: A Qualitative Study in Two Villages of Matlab

**DOI:** 10.3329/jhpn.v28i1.4528

**Published:** 2010-02

**Authors:** Nasima Selim

**Affiliations:** James P Grant School of Public Health BRAC University, 66 Mohakhali Commercial Area, Dhaka 1212, Bangladesh

**Keywords:** Culture, Depression, Perceptions, Qualitative studies, Bangladesh

## Abstract

This article reports the results of a qualitative study conducted in two villages of Matlab to explore the cultural dimensions of depression. Participants included adult men and women with and without a history of depressive episode (n=42), formal and informal healthcare providers (n=6), and caregivers (n=2). Adults (n=10) with a history of depressive episode were selected from a 2005 survey conducted by ICDDR,B. A case vignette was used for eliciting local terms for depression, perceived causes, impact, and treatments. Hardly anyone recognized the term *bishonnota* (literal translation of depression) used in the past survey. The participants thought that the vignette was about *chinta rog* (worry illness), and they spoke of somatic symptoms in relation to this condition. When explored further, they mentioned sadness and psychological complaints. Men felt that it affected them more while women felt the opposite. They associated *chinta rog* with poverty and social issues with impacts on marriage, work, and education. From their responses, it seemed that they preferred a psychosocial framework attributing the cause to thoughts and emotions, resulting from social causes. Commonly-suggested treatments were more income, better relationships, and tablets. Former health providers were often the first choice for help-seeking. The study hopes to ‘culturally inform’ the formal healthcare providers and programme planners.

## INTRODUCTION

Culture plays a significant role in shaping up depressive symptoms, its awareness and impact, and care-seeking. For instance, people with depression have more somatic symptoms in non-Western countries than their counterparts in the West (1). In Bangladesh, Chowdhury found a significant presence of somatic symptoms among 191 patients (2). Farooq *et al*. found more somatic and depressive symptoms among Asian patients (from India, Pakistan, and Bangladesh) compared to Caucasian patients in a primary-care setting of Britain (3). The sociocultural factors interact with the risk factors that contribute to depression.

For example, poverty and joblessness are known to intensify a sense of failure in certain cultures leading to depression (4). Cultural factors also affect diagnosis and management. It is important to understand the local term(s) and the popular concepts of the causes, effects of particular health problems, and help-seeking in the community if communication is to be improved between patients and healthcare providers (5, 6). Patients and their families may have their own ideas about the illness as opposed to clinicians’ views. ‘Emic’ (perceptions of the local community) and ‘Etic’ (perceptions of professionals) views are captured by the respective explanatory models (7). Anthropologists have extensively discussed a major difference among illness explanatory models, i.e. the physical framework that attributes illness to physical causes and the psychosocial framework that attributes illness to thoughts and emotions, usually resulting from social factors (8).

Studies in Latin America describe culturally-constructed idioms, such as nerves as the somatization of emotional distress, resulting from status deprivation (9). In Bangladesh, anthropological studies among the urban poor have described specific lay terms to describe emotional distress, such as *chinta rog* (worry illness), and associated it with various physical, emotional complaints and existential conditions of the entire body (10). The urban poor felt that socioeconomic deprivations caused their worries which resulted in various illnesses. Poverty and everyday sufferings were, thus, embodied and expressed through the body. In 2001, a study among rural women in Matlab found that the major reason for reported emotional stress was poverty (defined as the chronic deficit of daily necessities). Only non-poor women emphasized other reasons, such as family problems, death of a close relative, etc. (11). The study investigated the link between microcredit and emotional well-being but did not explore the ‘depression experience’ any further. Islam and colleagues found that people in urban settings did not perceive depression to be a mental disorder (12). Since there has been no such study done in a rural setting, we hardly know if this is also the case in the villages in Bangladesh. In fact, very little information is available on the perceptions of depression in rural Bangladesh. Published data from Bangladesh so far have focused on postnatal depression or depression as co-morbidity among patients with leprosy (13, 14).

In 2005, the International Centre for Diarrhoeal Disease Research, Bangladesh (ICDDR,B) conducted a non-communicable disease (NCD) risk-factor survey to collect data on depression in Block A and B of the ICDDR,B intervention area in the Matlab subdistrict, 65 km southeast of Dhaka, the capital city of Bangladesh. Personal communication with the ICDDR,B investigator of the 2005 survey revealed that the rate of depression was only 0.8%. In the survey instrument, depression was defined by the word *bishonnota.*

The usual practice of forcing the Western psychiatric diagnostic concepts to be recognized in non-Western contexts with literal translations may have methodological pitfalls (15). Sometimes, it is because the English word ‘depression’ may not be translatable to Bangla in terms recognized by the rural people in Bangladesh. Or, it could also be that subjective experience of depression, its manifestations, and the social responses to it differ across cultures (16). These dilemmas cannot be solved without first exploring the perceptions of ‘depression’ in rural settings. In response to these dilemmas and given the fact that the perceptions of depression in rural Bangladesh have never been investigated, the author conducted a qualitative study among adults in two villages of Matlab focusing on the various cultural dimensions of depression. The specific objectives of the study were to: (a) describe the illness experiences of people who had a previous depressive episode; (b) find out if the participants recognized depression from a typical case vignette; (c) identify the local term(s) for conditions similar to ‘depression’; (d) explore the perceived causes of depression; (e) detail the perceptions about the manifestations of depression; (f) find out the popular ideas about the prognosis and impact of depression; (g) describe the help-seeking behaviour of victims of depression; and, finally, (h) explore popular ideas about prevention and treatments of depression.

## MATERIALS AND METHODS

An exploratory, qualitative study was carried out among adults in two villages of Matlab, a subdistrict 65 km southeast of Dhaka. In-depth interviews and focus-group discussions (FGDs) were conducted with selected participants in these villages. The study was carried out for nine weeks from November 2006 to January 2007.

### Study site

ICDDR,B has a field research site in Matlab. The site is divided into four blocks for administrative and intervention purpose. The NCD risk-factor survey had collected data on depression about a year earlier in Block A and B of the ICDDR,B intervention area. Two villages—one each from Block A and B—were, therefore, selected conveniently for the current study so that the findings from these two very different settings within the same sub-district could be compared. Nabakalash is a geographically-accessible village in Block A, close to the main road just opposite to the Matlab Hospital of ICDDR,B. Results of a preliminary analysis of the 2005 survey data showed that mostly women in that village had depressive episodes. Nabakalash has a population of 2,580, with 57 livebirths per year (17). The government health centre is located within a short distance, and most formal health services are within reach. This village has a number of educational institutions, including high schools and colleges. By contrast, most people with a history of depressive episode in Nagda were men. Nagda, a fairly large village situated in Block B, has a population of about 4,424, with a high number (117) of livebirths per year (17). There is no high school or government healthcare centre nearby. The Matlab Hospital of ICDDR,B runs a subcentre with limited healthcare facilities. Nagda is representative of a remote rural area, with serious problems of access to essential health services.

### Study sample

Adults aged 25–64 years (matching the age-groups of the original survey) comprised the population from which purposeful sampling was done to select information-rich participants till redundancy was reached. Identification of information from the 2005 survey helped select participants who had a depressive episode that year. They were located using the Matlab Health and Demographic Surveillance (HDSS) of the ICDDR,B database. Participants were selected across gender, religion, various occupations, and socioeconomic status. For in-depth interviews, 10 adult men and women with history of depressive episode, two caregivers, three traditional healers (referred to as *kabiraj, fakir*, and religious healers, e.g. *Imams* who lead the Friday prayers and teach in local madrasa or religious schools), two village doctors [informal healthcare providers who have received training as paramedics in rural Bangladesh are commonly known as ‘*daktar*’ (transliteration of doctor)], an elderly villager, and an MBBS doctor working in Matlab were selected. Four FGDs—two in each village—were conducted with 31 men and women without a history of depressive episode.

### Data-collection procedure

A case vignette (Box 1) with typical features of a depressive episode was read out to each participant to initiate conversation (18). The original case vignette was translated into Bangla and modified with local names and idioms—by adding the description of a man for the men's FGD and that of a woman for the women's FGD. This particular vignette was used as a means of portraying depression without using technical language. It describes the behaviour of the person with an indication of age, sex, and main symptoms. Other studies have produced credible results by applying this case vignette (19).

**Box 1. Case vignette of depression**

Jomila/Rahim complains of different troubles at different times; troubles, such as headache, pain in the stomach, general weakness of the body, and tiredness. She/he may not be doing her/his work, as good as she/he usually could. She/he finds it difficult to sleep. In addition, she/he is worried about problems she/he faces (money, children, housing) and is irritable with close relatives and friends. She/he cannot relax or enjoy her/himself properly.

The following questions were asked after reading out the vignette: What do you think of Mr., Mrs. or Ms X? Does he/she have a problem? What kind of problem does he/she have? Is it an illness? Did you ever suffer from such illness? Did anyone in your family ever suffer? Do you know anyone in your village matching this description? Do you think this condition is physical, mental, both, or just social?

A few more questions from a published article by Wig *et al.* were adapted (17): What kind of manifestations does he/she have? Do you think there is a problem? If so, what would you call it? What do you think are the causes of his/her illness or problem? Is there anything that can be done about it? Who should do it? Do you think that this disorder will get worse? Do you think that this disorder is harmful to the individual, his or her family, or the community? How? Do you think this disorder will impair his/her chances of marriage, or the continuation of an existing marriage? Do you think that the individual will provoke problems in the home during his or her illness? How capable do you think the individual will be of working or studying? What kind of treatment do you think he/she should receive? What are the most important results you think he/she would receive from the treatment? Who do you think suffer from this condition the most? Do you have any suggestions about providing treatment for such problems? What do you think work best—prevention or cure? Do you think that this problem can be prevented and how? Additional questions for the traditional healers, village doctors, and the MBBS doctors were: What do you suggest to prevent such problems? What do you usually prescribe to treat such illnesses? Is there any ideal intervention? If so, what is that?

Each FGD comprised eight participants (only one FGD had seven people) and lasted for almost two hours. In the beginning of the discussion, after having read out the vignette, the participants were urged to collectively prepare a free-listing of all local terms that matched the condition described in the vignette. These discussions and the in-depth interviews were audio-taped. Notes of observations of the settings and the participants during each conversation and discussion were also kept by the author. This provided additional data.

### Analysis of data

The audio-taped materials were transcribed, and all data with additions from body-maps, free-listings, and observation notes kept by the author were compiled by the author. The Atlas.ti 4.1 software for Windows 95 was used for organizing, coding, and sub-coding the compiled data. ‘Data-reduction’ strategy was applied to focus on materials around the most common results with a few striking exceptions (20). For each question asked in the interviews and FGDs, ‘cross-case analysis’ was done (21). Answers from each participant were organized first according to the questions and then re-organized by gender, respective village, whether they were providers or clients, and finally according to whether they had a history of depressive episode or not. Later, emerging themes were identified and put in a logical sequence aimed at answering the research objectives.

### Ethical considerations

The Ethical Review Committee of the James P Grant School of Public Health approved the research proposal for this study. It was observed that participants were suspicious of written informed consents where they had to either sign or give finger stamps. Therefore, it was decided to obtain informed verbal consents with the help of a research assistant. Permission was also taken before audio-taping our conversations and discussions. All names have been converted into pseudonyms to ensure confidentiality. The participants were also assured that their comments would remain anonymous. Simple refreshments were provided during FGDs but no payment was made to any participant. Towards the end of the data-collection period, some participants (with a history of depressive episode) were advised to seek professional help from formal healthcare providers, e.g. MBBS doctors or, if possible, a specialist in psychiatry.

## RESULTS

### Profiles of participants

There were four women and a man with a history of depressive episode in Nabakalash while in Nagda it was the reverse: four men and a woman were found with a history of depressive episode from the past survey data. Except one, these women were all housewives. Men worked as rickshaw-pullers, scooter-drivers, farmers, land-owners, security guards, or shopkeepers. All of them were Muslims, and only one woman was Hindu. These men and women were married and had children. Only the scooter-driver in Nabakalash completed high school education. The remaining participants received little or no formal education. Two caregivers—one in each village—were interviewed to explore their perspectives, and an influential senior member of the community in Nabakalash was interviewed as well. He provided a lot of information.

The formal and informal healthcare providers were all Muslim men, except the Hindu *kabiraj* in Nabakalash. Dr. Mahmud, a 32-year old unmarried Muslim, was first interviewed. He was the MBBS doctor working in a government health facility in Nabakalash. He had been a few years junior to the author in the medical school. This facilitated our conversation and imbued it with a friendly spirit. In Nabakalash, *Imam* Hafez Belali, a 35-year old religious leader; *kabiraj* Ratheen Thhakur, a 40-year old herbalist; and a village doctor, the 45-year old Islam *daktar*, were also interviewed.

The author did not get the opportunity to meet with a formal healthcare provider in Nagda village. She interviewed *Kala huzur* (a holy man with dark complexion), a 70-year old widower and religious healer in the village, and a village doctor, a 41-year old man named Zahed *daktar*.

In Nabakalash, the female participants in the FGDs were aged 26–58 years, were more educated than the Nagda women, and belonged to a higher socioeconomic class. Nabakalash was a relatively-affluent village. Five women reported that their husbands worked outside Matlab—in Dhaka and Middle Eastern countries. The author met with a divorced woman in Nabakalash and a widow in Nagda. They seemed isolated from the remaining women who were apparently enjoying their full marital status. The female participants in the FGD were a little older than the women in Nabakalash, with most of them aged over 30 years. Only Sakhina had a job. She was a BRAC Community Health Volunteer (CHV), a *Shastho Shebika* (SS).

Men from Nabakalash were also more educated and belonged to a higher socioeconomic status than those from Nagda. All were married, except two young men in their mid-twenties. The age range of these men was from mid-twenties to mid-fifties. Men in Nabakalash were government employees, NGO workers, office assistants, rickshaw-pullers, and scooter-drivers, and one was a microbus-driver. Men in Nagda were mostly agricultural labourers, small traders, and rickshaw-pullers. The only unemployed man among them was from Nagda.

### Perceptions of depression in villages

#### Local terms

The participants described many local terms for the case vignette of depression, which are summarized in an ascending order (Box 2). Most agreed to what Mannan (aged 38 years, a scooter-driver patient) said, “*The problem starts in the mind, and it then affects the body.”* Others said that it was a physical problem. A few mentioned that it was just a social and financial problem. Mr. Huq was the only participant (other than the formal healthcare providers) who recognized the term *bishonnota* or depression. He considered it to be a mental disorder.

**Box 2. Local terms for depression**

*Chinta* rog (worry illness)
*Chinta* (worry)
Tension (anxiety)
Tension *rog* (anxiety illness)
Brain stop (brain stops functioning)
*Orthonoitik* (financial)
*Durbolota* (weakness)
*Mathaye* tension (tension in the head)
*Hridkampo/buk dhorfor* (palpitation)
*Hai hutash* (grievances)
*Manoshik rog* (mental disorder)

#### Manifestations of depression

The participants described a wide range of physical, emotional and social problems as manifestations of depression:

(Physical) Dizziness … sleeplessness … ache, and pain … burning sensation … do not feel like eating … cannot even move … stay in bed all the time … no strength in my legs … shaking limbs … cannot breathe … head is heated up … weakness … pressure from tension … heart problem.

(Psychological) Mind is never at rest … as if I have lost something … mind is full of thoughts … worry and tension. I become silent and feel hopeless; mind is full of problems; I get irritable; not just tension … worries about health problems … no peace … do not want to talk to anyone … I feel scared, as if someone is coming to get me.

(Socioeconomic) I have loans … I have daughters …. How am I going to marry them off …. Husband is old … who knows if he is able to earn today … son has no work; every day brings fresh worries. I have to worry about paying the installments every morning; what to eat? How shall I survive? Where from do I get money for dowry? … these are problems … real problems….

### Perceived causes

#### Poverty and depression

The participants mentioned poverty as the most common cause of such illness. One participant described:

Poverty is the reason. If you can meet your needs, the problems will lessen. Tension *rog* is more in these areas …. All tension *rogs* (anxiety illness) are due to *obhab* (want). (Mannan, a 38-year scooter-driver patient)

The village doctor from Nabakalash considered mental tension that came from poverty as the “*beginning of all diseases”.*

#### Girl child in the family

Rabeya (aged 44 years) is the mother of a son and three daughters from Nagda. Her husband works abroad. She lamented:

I have too many daughters … reason for my worries and tension—If I had sons, they would have an income. It costs a lot of money to get daughters educated. How shall I get them married off?

Having too many daughters was considered to be a major cause of worry and *chinta rog.* Daughters meant securing dowry money for her future, a constant source of tension among the older men and women whose daughters were now grown up. In both the villages, the women and elderly men complained of the dowry system. They said that, for families with too many daughters, dowry was the main reason why people were suffering from c*hinta rog.* This could either be a direct result of worrying about daughters or as a result of poverty that dowry brought to their home.

#### Physical and emotional ailments

The older men and women felt that their poor health and physical problems made them worry about the future. This was not a major issue for the younger people. Married women whose husbands lived abroad expressed loneliness and thought that this could cause *chinta rog*.

#### The spirits

Contrary to popular assumption about rural perceptions, most villagers were not at all convinced by ‘ghost stories’ or supernatural causes of depression. In most cases, they blamed the socioeconomic problems more than the unseen forces: *“This is from tension. This is not due to spirit or ghost ….”* (Women's focus group, Nabakalash)

#### Perceived prognosis and impact

According to the participants, the impact of depression was multiple, which affected work, education, and relationships. They expressed a grim future for the individual who had such illness. They felt that c*hinta rog* would worsen day by day if the person did not solve the problems that caused the condition. They felt that *chinta rog* could lead to other illnesses, such as stroke, heart attack, etc. They felt that sometimes people even committed suicide because of it.

A patient described the direct effects on his working abilities:

After a while, I cannot work. I become tired. I lie down, and I cannot do anything. This is my condition …. I go to work but I cannot work as much as is required. (Monowar, a shopkeeper and farmer from Nagda)

A caregiver complained of her husband's progressive impairment of functioning:

The whole world is involved in all sorts of work. He is the odd one out. He keeps on incurring losses. Because of his habits, he is destroyed. It is all in his mind. (Barkat's wife, Nagda)

The effect on relationships and marriage was discussed a lot. The participants mentioned that there would be no peace in the family, and marriages would be affected. For men, the question was about their capacities as men to provide support to the family. Women felt that they would get beaten if *chinta rog* had made them incapable of carrying out household chores.

It was not just a question of what impact depression had on marriage. The community stigmatized the illness and spoke openly against the marriage options of people with such illness. The logical connection was clear to them:

If someone is half-mad, who will marry him or her? No one will marry someone who has poor health. (Monir aged 36 years, an expatriate villager in Nagda)

Even if someone wished to marry such a person, he or she must have had some vested interest. According to them, such marriages would prove to be even more disastrous.

#### Perceived prevention

When the participants were asked about their opinions on how to prevent such illness, a few said that there was no prevention. They attributed the prevention to God's will or destiny. Some participants felt that higher education could prevent depression. Hard work was also suggested by some as a possible prevention. The participants who attributed the cause of depression to poverty felt that poverty alleviation could prevent depression. One participant mentioned government allowance to alleviate poverty which would eventually prevent depression. Another said that only a monarchy or autocracy could solve all economic and social problems with a lot of money, and there would be no depression. He said:

Money can solve all problems (Taka *hole shomoshya pratirodh shombhob*). (Saiful aged 38 years, an NGO worker)

#### Perceived treatment

The study participants felt that depression had no treatment (*chinta roger oshudh nai).* However, a few suggested that ‘poverty alleviation’, ‘peace in the family’, ‘good health’, ‘treatment for physical problems’, etc. could perhaps cure depression. Several participants talked about their own coping strategies, e.g. talking with others, going to the neighbour's house, getting involved in hard work, etc. They acknowledged the role of medicine but stated that it worked only to some extent and that it was not an ideal intervention. Saiful (an NGO worker) said:

A community-based programme is an ideal intervention. If I could form a collective *(somobaye somiti)*, especially to deal with this condition, if people could talk to each other and discuss their problems with each other, it would have been possible to solve the problems. Someone has a problem but others can come forward, and this crisis can be overcome.

Some participants felt that the social leaders should come together and discuss how to take care of these conditions with everyone.

Tablets seemed to be their preferred choice of treatment once the illness had progressed. It was only because they could not afford it and, as such, they did not take medication. The author had the opportunity to talk to a woman who had been suffering from depression for many years. She complained that she had no money to buy tablets. Nothing seems to have worked in these past years. So, she was keeping her hopes high on the tablets to cure her debilitating condition for many years.

#### Help-seeking practice

Although the participants in the study villages reported their preference for a specialist physicians or at least an MBBS doctor, it was at first surprising to hear that they did not always seek help from them. In reality, of course, they could not always afford to visit one. In Nabakalash and Nagda, the village doctors happened to be the most accessible healthcare providers, along with their more traditional counterparts—the religious healers and herbalists. In depression-like illnesses, only if they could not satisfy their clients or the conditions were getting worse, people would seek help from the local MBBS doctors. A typical pattern described by the participants was as follows:

Any affected person would first seek help from close relatives, family members, and, if necessary, the village elders to solve his/her immediate problems. If the problem could not be solved and the illness continued, they would consult either a traditional healer or a village doctor. If that did not work out, they would save or collect money to visit the nearby MBBS doctor or a ‘medical specialist’. No one ever mentioned going to a psychiatrist.

### Perceptions of health providers

#### Informal healthcare providers

The village doctors admitted the need for timely referral as a part of the ideal intervention of such illnesses. One of them was Mr. Islam who had worked for many years in an international organization as a field instructor. Afterwards, he got paramedical training from a private institute in Dhaka to become a village doctor. He owned a pharmacy and prescribed medicines for minor illnesses in the village. He did not recognize the vignette condition as depression but felt that it was a major problem that could affect the lives of people. He said that he treated people with such complaints symptomatically, with the available medicine in the pharmacy. Mr. Islam emphasized on the referral system and acknowledged the limitation of his role as a village doctor. He said that whatever the name of the problem was, it was a mental problem and needed to be treated carefully. In his opinion, the social problems and financial worries should be solved socially and the physical symptoms physically with medicines.

Mr. Zahed, a village doctor in Nagda, also owned a pharmacy and ran a grocery shop under the same roof. He recognized the vignette condition as *chinta rog*. He said that it was a temporary problem due to tension or other physical illnesses. He did not think that it was a major problem and felt that it could be solved within the community. He reported that he provided short-term relief, e.g. sleeping tablets, ‘mixed tablets’ (anti-depressants with anti-psychotics), and anxiolytic drugs from his pharmacy. He believed that tablets can provide the cure. If the patients did not get better, he referred them to the MBBS doctor.

#### A healer drives away evil spirits

In Nagda, the author met with an elderly religious healer who called himself *Kala huzur.* He was a dark-skinned man, dressed in a grey coat with a long white beard. He stated that he was a *kabiraz* (a herbalist) and a *maulavi* (a Muslim learned man) because he could heal people and was also involved in teaching young students in the local *madrasa* (religious school). He explained the vignette conditions with great confidence. He said that the condition was caused by *jalishtir deo*. According to him, a *jalishtir deo* was the soul of a *thief, a rogue, a scoundrel, and jinn.* He mentioned that 99% of the people had it, which was obviously an exaggeration. He felt that women were more affected by it. His treatment for the condition was also plain and simple. He advised his amulets and enchanted oil for 40 days and uttered Quranic verses. In his opinion, this was the only effective treatment available.

Ratheen, a traditional healer in Nabakalash, stated that he did not deal with depression at all. He told that there were specialists within their discipline as well. He only gave treatment for different kinds of pain, asthma, and diarrhoea but he was well-informed about the other traditional ‘specialists’ who dealt with the conditions the researcher was interested in. He spoke of a religious healer in the other side of Matlab. He said that Ishaq *huzur* across the river was the ‘*pagal*’ (madmen) specialist who also treated *chinta rog* but Ishaq *huzur* saw patients only during his prayer time, and only men could access him. There was no way he would meet with a woman. Since the author was a woman and could hardly pose as a male disciple, she had to abandon the plan to interview him.

#### Formal healthcare provider

The MBBS doctor said that there was no major difference in the presentation of symptoms between people from Matlab and urban people. He felt that most patients in Bangladesh usually reported somatic complaints, e.g. loss of appetite, sleeping problem, multiple pain, and burning sensations. Once the doctor had paid attention to these symptoms, the patients would describe the mood problems, irritability, sadness, and behaviour changes. He thought that the case vignette described both depression and anxiety, which, according to him, is most common in this part of the world. He also mentioned family problems (e.g. too many daughters) as a major cause of worry for parents. He felt that suicide was common among patients with depression.

Dr. Mahmud mentioned that, although people still preferred traditional healers and village doctors, the women were seeking help from the MBBS doctors more than ever before. He could not specify why but said that it could be because the younger and more educated women had trouble explaining their psychological sufferings to the traditional healthcare providers in the village. They probably preferred outsiders, such as doctors from the city, to retain relative anonymity. The doctor felt that the prevention and treatment of ‘depression and anxiety’ should be multi-pronged, with a combination of drugs, counselling, and family-focused interventions.

## DISCUSSION

### Underestimation of depression and its probable impact

On the whole, it seemed that the villagers strongly felt that the problem described in the case vignette was widespread. The objective measure of depression (0.8% according to the ICDDR,B survey) did not seem to suggest that it was a major burden in Matlab or in these villages. Yet, the perceptions of the villagers, the healthcare providers, and especially those with a history of depressive episode provided a picture of widespread suffering. It may be depression, anxiety, or both. It is, after all, a matter of subjective well-being, and participants in this study clearly provided a dismal picture.

Besides, almost nobody recognized the word *bishonnota,* which means ‘depression’ in the survey questionnaire. It can be argued if depression was under-reported by that survey simply because the word defining the condition was too alien and unfamiliar in rural Bangladesh. Could the survey results be different if, instead of local term *bishonnotaa*, the ones freelisted by the inhabitants were used for defining ‘depression’ in Matlab.

However, if depression continues to be under-reported, it could have serious consequences on health and productivity. An underestimation may lead to little or no intervention with much less priority from health planners so that eventually it may claim lives. The rural community expressed a similar concern when they said that *chinta rog* could lead to death by suicide. The perceptions of the community are in keeping with the scenario across South Asia. In South India, suicide is one of the leading causes of death among young women (22). People have talked extensively about the impact of depression on work and family life. These findings are consistent with a large body of evidence, indicating the disabling effects of depression in the community (23).

### Poverty and depression

The majority perceived poverty to be related to *chinta rog.* Studies have found that persons at the bottom-end of social hierarchy are at a greater risk of suffering from depression (24). Although Islam found mental illnesses (including depression) to be more common among the higher socioeconomic classes in the urban setting, he speculated that perhaps people of the poorest section were so concerned with immediate economic survival that they did not consider the socio-political, familial or social stresses that the upper classes were preoccupied with (25). Besides, the findings of Islam could not have been representative of rural Bangladesh as it was conducted in cities.

Patel and colleagues found that lack of education (a strong correlate of poverty) was strongly associated with depression in many developing countries (26). Popular perception did not implicate education but some thought that lack of it could cause more *chinta rog* (worry illness). Since higher education can determine present and future opportunities or ensure income-generation, it is plausible that this might work towards preventing depression in later life.

Situating the perceptions of depression in rural Bangladesh within the broader context of the available quantitative measurements of depression and its correlates in developing countries, like Bangladesh, provides a broader picture with which policy-makers can design more informed health measures in the community. It also stimulates the thinking about how to make the two ends meet, especially in situations where perceptions and objective measurements do not match.

### Gender and depression

The impact of the author's gender status was obviously more extensive on the male participants causing their initial hesitation in responding to her questions. There was also an evident gender bias about depression among the participants. Men felt that depression or *chinta rog* was more common among men whereas women felt that it was the women who mostly suffered from it. Perceptions of women seem to be consistent with results from Bangladesh (27) and other developing countries where an average of 6–10% of women suffer from the condition, and much higher rates have been found among women attending primary healthcare centres (28, 29). Contrary to popular belief or even objective measurements, men perhaps do not suffer less. It may well be that the cultural pressure on Bangladeshi men to become *macho* men (men with a stereotypical image of invincible masculinity) discourages them to self-report or seek help. Men in this study described a great deal of sadness and anxiety relating to their roles as the main support providers to their families. This might have driven those with depression to seek relief in either smoking, or to other kinds of substance abuse.

### Explanatory models of depression

As mentioned earlier, most anthropologists have discussed that the major difference in the explanatory frameworks of illness is the difference between the predominantly ‘physical’, i.e. attributing illness to only physical causes and the predominantly ‘psychosocial’ models, i.e. attributing illness to one's or others’ thoughts and emotions resulting from social causes (8). In most cases, ‘biomedicine’ opts for a physical explanatory model, and the people in non-Western cultures prefer a psychosocial model that provides a far richer understanding than the mechanical cause-and-effect explanation offered by biomedical practitioners (8).

In the present study, the villagers and informal healthcare providers perceived ‘depression’ with a ‘psychosocial’ explanatory model. They believed that, having too many *chinta* (worries) could lead to *chinta rog* (worry illness) that resembled the typical description of a depressive episode. It is felt that the case vignette in this study used did not have any medical jargon but succeeded in eliciting wider recognition and responses from the community.

Patel has argued against using psychiatric terms in some less-developed countries, particularly labels, like ‘depression’, because such terms had little meaning or value in non-Western settings. He suggested that the use of locally-valid terms and idioms would rather enhance recognition (29). It can be argued, however, that the vignette used included many more somatic symptoms than the psychological, and it, thus, might have biased the participants towards reporting more somatic symptoms in relation to such illnesses. It was tried to get over this initial bias by later pursuing and exploring in depth if they really felt it to be so or if they were also concerned with the psychological complaints. This effort had some results because many of them actually spoke of psychological symptoms which resembled those of depression.

### Patterns of distress

Most participants described somatic complaints, e.g. multiple aches and pains, sleeplessness, burning sensations, weakness, etc. and associated these with terms, such as *chinta rog,* or tension *rog*, the most commonly-cited terms that matched description in the vignette. The participants drew body-maps that clearly showed that they recognized, and emphasized on, the association of physical problems with *chinta rog.* This predominance of somatic complaints in depression is consistent with the results of other quantitative (2) and qualitative (10) studies that investigated the symptom profile of depression and psychological suffering among the urban population. It may well be that physical complaints are culturally more acceptable than emotional symptoms, as often seen by the professional medical doctors in this study. The fact that the participants began with physical complaints only to elaborate on their psychological complaints with just a little probing confirms the findings of other researchers in both Western and non-Western settings (15, 30).

### Perceived causes and remedies

The participants associated depression with *worries … too much worries.* It was seen as a chronic condition that resulted from financial worries, health problems, family issues, etc. However, the socioeconomic situation was seen as a major causal factor by all the participants. Therefore, the suggested remedies were also focused on improving the financial condition. It was interesting to note that microcredit was implicated both as a cause and a remedy. While conservative men thought of it as a social curse and financial catastrophe, for some women this was a life-saver. However, results of previous studies in rural Matlab suggested that only microcredit was not sufficient to ensure the women's emotional well-being (11). The comments made by the respected elders raise thought for concern. If the community is threatened by women's empowerment, even with economic improvement, the women would not be necessarily happier. Interestingly enough, belief in supernatural cause seems to be less prevalent in Matlab. Only one isolated response from a religious healer implicated bad *jinns* (evil spirits). However, the researcher feels that many participants might have thought that she did not like to listen to ghost stories. Sufficient probing of the negative responses might yield a different result.

A few suggestions about community-based interventions, e.g. a place where the villagers can share their concern and grievances are valuable for designing future interventions but this probably requires further investigation.

### Help-seeking

Help-seeking in this rural community showed a specific pattern. There are preferred and actual sources of help within the community. Religious and traditional healers were rarely the preferred choice. It seemed that the participants preferred specialists and MBBS doctors but, due to financial and geographical inaccessibility, they settled for the village doctors and local healers. Somatic complaints could also explain such preferences. The community felt that MBBS doctors and specialists could do more about relieving physical complaints. In a cultural epidemiological study in Bangalore, Raguram and colleagues found similar preferences among patients presenting for the first time in a psychiatric clinic (15). Participants with a history of depressive episode and treatment, however, expressed their dissatisfaction about treatment. They preferred tablets that could cure all but, due to financial constraint, they could not often buy medicine. Biomedically trained physicians are often concerned with their own professional concepts of disease and speak the standard medical jargons. Instead of explaining the disease in local terms, or making efforts to initiate a ‘culturally-informed inquiry’ into the embodied experience of their patients, they often become inattentive, if not openly abusive, to the patients’ sufferings. Excessive reliance on the universalistic accounts of psychiatric disorders can further marginalize the cultural considerations (15).

### Local insiders versus professional outsiders

The villagers and the informal healthcare providers expressed commonly-held views. They are the ‘local insiders’ whose views differed from the formal healthcare providers who came from outside. The MBBS doctor and the psychiatrist comprise the ‘professional outsiders’ (7). The terms used by the insiders (*chinta rog,* tension *rog,* etc.) were different from these outsiders who used medical jargons (major depressive episode, depression and anxiety, etc.) to describe the case vignette explaining a depressive episode. There was, however, agreement on financial problems causing a lot of *chinta* (worry) and in treatment modalities suggested by the professionals. The community preferred tablets if there was a chance to be cured soon, although their focus was on ‘problem-solving’. In their opinion, the socioeconomic problems were responsible for *chinta rog.* Although the village doctors were more in favour of modern treatment, the traditional healers preferred amulets and trust in Allah. This raises the question if it is possible to form a liaison among formal and informal healthcare providers. The community was divided into more- and less-conservative group of people. The village doctors and less-conservative members of the community could, however, act as entry points into setting up of acceptable and accessible community-based services.

### Limitations

The author had a prior assumption that depression or its literal Bangla term—*bishonnota*—was not a well-recognized term, and, therefore, felt, as Kreuger had said, that it could be possible to get better-quality data by illustrating the topic (instead of focusing on a little-known term) (31). Presenting a same gendered description may have biased the participants from the beginning. Ideally, everyone should have been presented with a vignette using both male and female case description. However, this was not possible due to time and budget constraints.

Within the stipulated study period, it was just possible to do a preliminary exploration of the topic. Only two villages from the two blocks were covered. Inclusion of a few more villages with varied characteristics could have added more richness and depth to the data.

Finally, and most importantly, data for this qualitative study were collected after more than a year after the original survey had been conducted. Those who had suffered from a depressive episode in 2005 might not be suffering from similar conditions at the time of the current study period. Important information may have been lost because of their trouble to recall how they were in 2005.

The present study explored the cultural dimensions of depressive episode in only two villages in rural Bangladesh. It provides us with previously-unexplored rural perceptions of depression, its causes and perceived treatments, and help-seeking behaviour of those who suffered from similar conditions. Based on the results of this study and further studies, interventions can be designed in combination of emic and etic perspectives on depression. For example, a survey could use local terms instead of an ‘etic’ term not commonly recognized by the villagers. The programme planners could also explore the existing coping strategies and focus on strengthening these further. The author's intention is to ‘culturally inform’ the formal healthcare providers and programme planners and propose that joint initiatives involving the ‘local insiders’ and the ‘professional outsiders’ can perhaps lead towards acceptable and affordable bio-psychosocial interventions for depressive illness in rural Bangladesh.

## ACKNOWLEDGEMENTS

The study was supported by the James P. Grant School of Public Health and ICDDR,B. This paper is based on part of the author's dissertation in partial fulfillment of the Master of Public Health (MPH) degree at BRAC University. The author is grateful to her supervisors for guiding her. She also thanks ICDDR,B for providing data from the original survey for selection of participants and for sharing preliminary results.
